# Biocompatible Coatings from Smart Biopolymer Nanoparticles for Enzymatically Induced Drug Release

**DOI:** 10.3390/biom8040103

**Published:** 2018-09-28

**Authors:** Christian Tolle, Jan Riedel, Carina Mikolai, Andreas Winkel, Meike Stiesch, Dagmar Wirth, Henning Menzel

**Affiliations:** 1 Institut für Technische Chemie, Technische Universität Braunschweig, Hagenring 30, 38106 Braunschweig, Germany; ctolle1@aol.com; 2 Helmholtz-Zentrum für Infektionsforschung, Inhoffenstrasse 10, 38124 Braunschweig, Germany; jan.riedel@helmholtz-hzi.de (J.R.); dagmar.wirth@helmholtz-hzi.de (D.W.); 3 Medizinische Hochschule Hannover, Carl-Neuberg-Str. 1, 30625 Hannover, Germany; mikolai.carina@mh-hannover.de (C.M.); winkel.andreas@mh-hannover.de (A.W.); stiesch.meike@mh-hannover.de (M.S.)

**Keywords:** smart drug delivery, ionotropic gelation, nanogel, enzymatic cleavage, cyto-compatibility, cell adherence, alginate, X-ray photoelectron spectroscopy, reflection-absorption infrared spectroscopy

## Abstract

Nanoparticles can be used as a smart drug delivery system, when they release the drug only upon degradation by specific enzymes. A method to create such responsive materials is the formation of hydrogel nanoparticles, which have enzymatically degradable crosslinkers. Such hydrogel nanoparticles were prepared by ionotropic gelation sodium alginate with lysine-rich peptide sequences—either α-poly-L-lysine (PLL) or the aggrecanase-labile sequence KKKK-GRD-ARGSV↓NITEGE-DRG-KKKK. The nanoparticle suspensions obtained were analyzed by means of dynamic light scattering and nanoparticle tracking analysis. Degradation experiments carried out with the nanoparticles in suspension revealed enzyme-induced lability. Drugs present in the polymer solution during the ionotropic gelation can be encapsulated in the nanoparticles. Drug loading was investigated for interferon-β (IFN-β) as a model, using a bioluminescence assay with MX2Luc2 cells. The encapsulation efficiency for IFN-β was found to be approximately 25%. The nanoparticles suspension can be used to spray-coat titanium alloys (Ti-6Al-4V) as a common implant material. The coatings were proven by ellipsometry, reflection-absorption infrared spectroscopy, and X-ray photoelectron spectroscopy. An enzyme-responsive decrease in layer thickness is observed due to the degradation of the coatings. The Alg/peptide coatings were cytocompatible for human gingival fibroblasts (HGFIB), which was investigated by CellTiterBlue and lactate dehydrogenase (LDH) assay. However, HGFIBs showed poor adhesion and proliferation on the Alg/peptide coatings, but these could be improved by modification of the alginate with a RGD-peptide sequence. The smart drug release system presented can be further tailored to have the right release kinetics and cell adhesion properties.

## 1. Introduction

Stimuli-responsive release strategies are expected to have a major impact on the development of innovative drug delivery systems (DDS) [1]. The application of nanomaterials as drug carriers has been shown to increase drug stability, reduce drug resistance, and even enhance therapeutic efficacy [2]. Moreover, drug-loaded nanoparticles (NP) with controlled release mechanisms offer the great advantage that a single dose can sustain therapeutically effective drug levels over a long period. Stimuli-responsive controlled drug release systems have been published by several groups. Amongst these are drug carriers responding to internal stimuli, such as pH, ionic strength, and biomolecules. Drug delivery systems can be also responsive to external stimuli, like ultrasound, light, magnetic fields, or chemical agents [3,4,5,6,7,8,9,10,11]. Herein, we report on the possibility of utilizing biomolecules like enzymes to trigger a specific drug release. Enzymes are critically involved in many biological and metabolic processes, representing a variable toolbox to work with. Thus, enzymes pose an attractive opportunity to catalyze chemical reactions under mild conditions, like low temperature, moderate pH, and buffered solutions [12,13,14]. Furthermore, their remarkable selectivity towards their substrates paves the way for innovative drug delivery systems [14,15].

The fabrication of nanomaterials exhibiting certain biomolecule responsiveness has already been studied. The most prominent strategy includes the preparation of enzyme labile carriers that are stable in absence of a corresponding enzyme and will be degraded when it is present [13,15]. Preferentially, the considered enzymes are secreted as a result of a disease to immediately trigger the drug release, which then helps to overcome the pathogenic situation. Such intelligent drug delivery systems are preferentially prepared from a biopolymer scaffold that is degradable itself, or from a polymer and a degradable linker. Baier et al. have fabricated polyhexanide-loaded hyaluronic acid nanocapsules using the inverse miniemulsion technique [16]. These capsules are degraded when the inflammation-related hyaluronidase is present and leads to a drug release. Radhakrishnan et al. developed dual-responsive microparticles based on the arginine-rich protein protamine and the biopolymer chondroitin sulfate. The degradation and subsequent drug release have been performed in the presence of infection-relevant enzymes, either trypsin or hyaluronidase [17]. Similar approaches have been pursued by the introduction of specific peptide sequences that serve as a crosslinker for the polymer matrix. In this way, the corresponding cleavage site can be simply tailored through peptide synthesis to adjust directly the release properties, depending on the enzyme. This strategy has been used by Seliktar et al., who introduced a thiol-endcapped metalloproteinase labile peptide sequence to crosslink polyethylene glycol hydrogels [18]. Fuchs et al. have fabricated nanoparticles based on polystyrene–peptide–polystyrene triblock polymers. By incorporation of an enzymatically degradable peptide middle block, they were able to demonstrate the release of a fluorescent dye upon addition of trypsin and hepsin [19].

Our approach is based on the preparation of nanogels through ionotropic gelation of the biopolymer sodium alginate with a specific peptide crosslinker, and subsequent coating of implants with this nanogel dispersion. The biodegradable and biocompatible alginates, extracted from brown algae, are extremely versatile biomaterials with manifold applications in the field of biomedicine [20]. The use of the biopolymer sodium alginate as a matrix for the encapsulation and delivery of biomolecules like proteins, DNA, or cells has been intensively studied [21,22,23]. The biological activity of guest molecules is widely preserved due to the relatively mild gelation in the presence of counter ions and the avoidance of organic solvents [24]. Enzyme-labile alginate hydrogels can be prepared by introducing specific peptide sequences as a complexation partner. Fonseca et al. transformed sodium alginate into a three-dimensional cell culture microenvironment equipped with enzyme-labile peptide sequences. The lability against matrix metalloproteinases (MMPs) helped to prepare degradable alginate networks for tissue engineering [25].

Due to their negative charge in aqueous media alginates, they can be used to form nanoparticles in combination with an appropriate multivalent cation (e.g., calcium ions) [26]. Here we studied the particle formation properties of alginate with two lysine-rich and therefore cationic peptides, namely α-poly-L-lysine (PLL) or an aggrecanase-labile sequence (KKKK-GRD-ARGSV↓NITEGE-DRG-KKKK) equipped with an additional tripeptide spacer and four lysine residues on each side. The protease aggrecanase belongs to the family of ADAM (a disintegrin and metalloprotease) proteins. It is mainly secreted during the early stages of inflammations—for instance, in osteoarthritis—and leads to the degradation of cartilage [27,28]. The nanoparticles were formed through an ionotropic gelation process based on electrostatic interactions, since this method is a promising tool for encapsulation of proteins [29]. The particles were characterized with regard to particle size by means of dynamic light scattering (DLS) and nanoparticle tracking analysis (NTA). The stability of the colloidal systems was investigated by zeta potential measurements. The nanogel dispersions can be used to coat implant surfaces, for example, by spray coating in order to obtain bioactive surfaces or antibacterial surfaces, which can release growth factors [30] or antibacterial substances. As a model for an implant, we used polished plates of a commonly used titanium alloy (Ti-6Al-4V). The characterization of the layers has been performed by ellipsometry, reflection-absorption infrared spectroscopy (RAIRS), and X-ray photoelectron spectroscopy (XPS). With regard to developing a DDS, we designed a biodegradable system to include hydrophobic proteins, such as interferon-β (IFN-β), which possesses antiproliferative and immune modulatory therapeutic effects [31].

## 2. Materials and Methods

### 2.1. Materials

Polyethyleneimine (PEI) was obtained from Sigma-Aldrich (St. Louis, MO, USA) and diluted to 5% (v/v) with MilliQ water. Poly-L-lysine hydrochloride (PLL, molecular weight 1.6 kDa, equals 12–13 lysine units) was bought from Alamanda Polymers (Huntsville, AL, USA). The aggrecanase-labile peptide sequence “KKKK-NITEGE↓ARGSV-KKKK-carboxyl” (NITEGE) was prepared at the Helmholtz Centre for Infection Research (Braunschweig, Germany), according to standard solid-phase peptide synthesis protocols. All solutions were filtered through 0.22 µm Millex–GP (polyethersulfon; Sigma-Aldrich) filters before use. Glycosylated IFN-β was purchased from PBL Interferon Source (Piscataway, NJ, USA) and was treated according to the manufacturer’s instructions. Trypsin, thermolysin, and alginate-lyase were all obtained from Sigma-Aldrich and used as received.

Titanium alloy substrates (1 mm thick, Goodfellow, Hamburg, Germany) were cut into 1 × 1 cm² squares and treated as described below. Acetone, methanol, ethanol, and dichloromethane were distilled before use.

### 2.2. Alginate Purification

Sodium alginate (Alg, average molecular weight 80 kDa) was purchased from Sigma-Aldrich. The purification was performed according to the protocol published by Prokop and Wang with slight changes [32]. Briefly, sodium alginate was dissolved in deionized water (1 g alginate/15 mL water) and was dialyzed (10 kDa MW cutoff) for three days, with continuous water exchange. Afterwards, the solution was stirred at room temperature with 0.5 g activated charcoal per gram sodium alginate, filtered through a Buchner funnel, lyophilized, and stored at −20 °C until use.

### 2.3. Ionotropic Gelation

The Alg/PLL and Alg/NITEGE-nanoparticles were prepared according to the following general procedure. Purified sodium alginate was dissolved in MilliQ water to obtain a final concentration of 1 mg/mL. After complete dissolution, a 1 mg/mL PLL or NITEGE solution was flush mixed with the alginate solution. Nanoparticles are formed spontaneously by electrostatic interaction between anionic alginate molecules and cationic peptides. Particle characterization with regard to particle size and zeta potential was performed right after preparation.

For encapsulation of IFN-β, ionic gelation was carried out with only slight modifications to the abovementioned protocol. An IFN-β stock solution was diluted to a concentration of 5000 relative light units (RLU) per mL. Subsequently, a defined volume of sodium alginate solution (1 mg/mL), was pipetted into a flask before the required volume of IFN-β solution was added. Nanoparticles then spontaneously formed upon addition of PLL or NITEGE solutions (1 mg/mL), respectively. The encapsulation efficacy was determined to be around 25%. The nanoparticle suspensions obtained were characterized for each batch by dynamic light scattering.

### 2.4. Particle Size and Zeta Potential

Particle size and zeta potential measurements were carried out using a Zetasizer Nano ZS from Malvern Instruments (Malvern, UK). Disposable sizing cuvettes (DTS0012) were used for size measurements, and clear disposable zeta cells (DTS1060C) for zeta potential measurements. All measurements were performed at 20 °C except the particle degradation experiments, which were conducted at 37 °C. Malvern Zetasizer Software Version 7.03 was used for data evaluation.

### 2.5. Nanoparticle Degradation

The degradation of the Alg/peptide nanoparticles was carried out through the addition of trypsin, thermolysin, and alginate-lyase to the nanoparticle dispersion. Briefly, 990 µL Alg/peptide nanoparticle suspensions (1 mg/mL) were filled in a Zetasizer sizing cell and maintained at 37 °C. Different enzyme solutions (trypsin, thermolysin, alginate-lyase) were added in order to obtain final concentrations of 2.5 µg/mL. The degradation process was then monitored via consecutive size measurements using the Zetasizer Nano ZS.

### 2.6. Nanoparticle Tracking Analysis

Alg/peptide suspensions were prepared according to the standard procedure and subjected to NTA using Nanosight NS300 equipment and NTA 3.1 software (both from Malvern Instruments) for the data analysis to obtain the diameter of particles. The suspensions were diluted 1:10 in MilliQ water prior to the measurement. The images were captured using a sCMOS camera at camera level 15, slider shutter set at 1206, slider gain at 366 and frames per second at 25. 1498 frames were captured and analyzed at a detection threshold set to 8, and max jump distance set to 11.2 pixels.

### 2.7. Bioluminescence Measurement in Solution

Mx2Luc2 cells were seeded in 24-well cell culture plates with 5 × 104 cells per well and incubated overnight at 37 °C. The next day, the Dulbecco’s modified Eagle’s medium (DMEM) was removed before IFN-β in medium (concentration series) or the supernatant of the centrifuged Alg/NITEGE/ IFN-β (encapsulation efficiency measurement) was added. The treated cells were incubated overnight at 37 °C before the supernatant was removed. Each well was washed with phosphate buffered saline (PBS) and treated with 125 µL reporter lysis buffer (RLB). The 24-well plates were stored in a refrigerator at 80 °C for at least 30 min. In a tube, 20 µL of each well was pipetted into 100 µL of luciferin solution and subsequently measured with Lumat LB 9507 by Berthold Technologies (Bad Wildbad, Germany).

### 2.8. Bioluminescence Measurement from Coated Titanium Substrates

A 20 µL solution of IFN-β-loaded Alg/peptide nanoparticles was coated onto PEI-modified titanium samples. After rinsing with water, the titanium samples were incubated in different media, including DMEM, DMEM with fetal calf serum (FCS), or PBS. After different periods of time, the supernatants were analyzed by the bioluminescent assay as described before. 

### 2.9. Surface Coating

The modification of Ti-6Al-4V substrate surfaces with Alg/peptide nanoparticles was carried out following a general procedure: The substrates were grinded, polished, and cleaned through ultrasonification in distilled dichloromethane, acetone, methanol, and MilliQ water. The washed samples were plasma cleaned and subsequently used for surface coating. Firstly, the substrates were immersed for 1 min in an aqueous 5% (v/v) PEI solution before rinsing in water for 15 s. The PEI-coated substrates were then spray-coated manually with an airbrush Aztek A470 from Testors (Vernon Hills, IL, USA) for 3 min, depositing approximately 20 µL of the Alg/peptide nanoparticle formulation. Afterwards, the substrates were rinsed in MilliQ water and dried in a stream of nitrogen.

### 2.10. Ellipsometry

Layer thicknesses were determined using a Multiskop from Optrel (Sinzing, Germany) in the ellipsometry mode. Uncoated titanium plates served as a reference for the corresponding coated samples. Data were collected in the x,y-mode at 70° as mean value of 16 data points in total. Evaluation of the data was carried out using Elli Version 3.2 from Optrel. 

### 2.11. Reflection-Absorption Infrared Spectroscopy

Reflection-absorption infrared spectroscopy spectra on coated titanium plates were recorded using a Bruker Equinox 55 FTIR spectrometer (Bruker Optik GmbH, Ettlingen, Germany) equipped with a Bruker A518 RAIRS module reflecting the infrared beam at an angle of 80° to the MCT detector. Background measurements were carried out with uncoated substrates in the range of 4000–400 cm^−1^, at 10 KHz and 32 scans before each coated sample was analyzed (4000–400 cm^−1^, 10 KHz and 64 Scans). Instrument control and initial data processing were performed using Opus software (Version 4.0.24) from Bruker (Billerica, MA, USA).

### 2.12. X-ray Photoelectron Spectroscopy

X-ray photoelectron spectroscopy measurements were carried out using the X-ray photoelectron spectrometer Axis Supra from Kratos Analytical (Manchester, UK), equipped with a monochromatized Al Kα X-ray source. Survey scans were acquired using a pass energy of 160 eV. High-resolution spectra (C1s, O1s, N1s, and Ti2p) were obtained using a pass energy of 20 eV. An area of 700 µm x 300 µm of each sample was analyzed with a power of 300 W, applying a charge neutralizator for 2 min.

### 2.13. Cytocompatibilty of the Nanoparticle Suspension

Cytocompatibility was tested with human gingival fibroblasts (HGFIB, Cat No.: 121 0412), which were purchased from Provitro GmbH (Berlin, Germany). The cells were cultured in DMEM (FG0435, Biochrom AG, Berlin, Germany) supplemented with 10% FCS (P270521, PAN-Biotech GmbH, Aidenbach, Germany), 100 U/mL penicillin, and 100 μg/mL streptomycin (A2212; Biochrom AG, Berlin, Germany). The cells were incubated at 37 °C in a 5% CO_2_, 95% humidified air atmosphere.

The cytocompatibility of the nanoparticle suspensions was studied by determining the cellular membrane damage and metabolic activity of human gingival fibroblasts. Human gingival fibroblasts were seeded in 96-well cell culture plates at a density of 1.5 × 10^4^ cells/mL and precultured for 24 h. The alginate nanoparticle suspensions were diluted in DMEM supplemented with 5% FCS, 100 U/mL penicillin, and 100 μg/mL streptomycin. The nanoparticles were diluted to different concentrations of 12.5, 25, 50, 100, and 200 µg/mL to represent the possible amount in the coatings. MilliQ water was diluted in the same way as a control. The suspensions (200 µL) were added to the precultured cells. After 24 h, the cellular membrane damage was determined by detecting the amount of lactate dehydrogenase (LDH) which had leaked the inside of the cells. The cell culture supernatant (100 µL) was mixed with 100 µL of staining solution (Cytotoxicity Detection Kit, 11644793001, Roche, Basel, Switzerland) and incubated for 10 min in the darkness. The reaction was stopped with 50 µL of 1 M HCl. Absorbance was measured with a multiplate reader (Infinite F200, Tecan Group Ltd., Männedorf, Switzerland) at 490 nm (reference 690 nm). For analysis of the metabolic activity, 20 µL of the CellTiter-Blue^®^ Reagent (CellTiter-Blue® Cell Viability Assay, G8081; Promega, Madison, WI, USA) was added to the cultured cells (100 µL). After 4 h incubation at 37 °C in a 5% CO_2_, 95% humidified air atmosphere fluorescence (540 Ex/595 Em) was detected with a multiplate reader (Infinite F200).

### 2.14. Cell Adhesion and Proliferation

As described previously, a modified LDH activity assay was used to evaluate the HGFIB adhesion and proliferation on the nanoparticle coatings in comparison to uncoated titanium, according to Heuer et al. [33]. Briefly, for the adhesion/proliferation testing, the coated and uncoated titanium discs were placed in 24-well cell culture plates and were covered with human gingival fibroblasts at a density of 1.5 × 104 cells/mL/well. After an incubation period of 24 h or 72 h, the samples were washed with Hanks’ Balanced Salt solution (HBSS) to remove unattached cells. The adherent cells were lysed using 10% Triton-x-100 (93416, Sigma-Aldrich Chemie GmbH, Steinheim, Germany) at 37 °C for 30 min. The released lactate dehydrogenase was measured with the Cytotoxicity Detection Kit. The number of adherent fibroblasts was calculated through comparison with a corresponding standard curve. The statistical evaluation of the cell adhesion and proliferation were performed using GraphPad Prism 7 (GraphPad Software, La Jolla, CA, USA). A Kruskal–Wallis test was used to analyze the statistical differences between the uncoated and coated titanium. Differences were considered statistically significant at *p* < 0.05.

### 2.15. Cell Morphology

Titanium samples were rinsed with PBS (L1825, Biochrom AG) and fixed for 4 h in 0.1% glutaraldehyde and 4% paraformaldehyde diluted in 200 mM HEPES buffer (4-(2-hydroxyethyl)-1-piperazineethanesulfonic acid from Sigma Aldrich). Afterwards, the samples were dehydrated in graded ethanol solutions before being dried completely through critical point drying. Samples for scanning electron microscopy (SEM) were mounted on stubs, sputtering coated (POLARON Sputter Coater SC7500, Ringmer, UK) with a thin layer of gold and measured in a SEM 505 (Philips, Eindhoven, The Netherlands) at 10 kV.

## 3. Results and Discussion

### 3.1. Ionotropic Gelation

**Figure 1:**
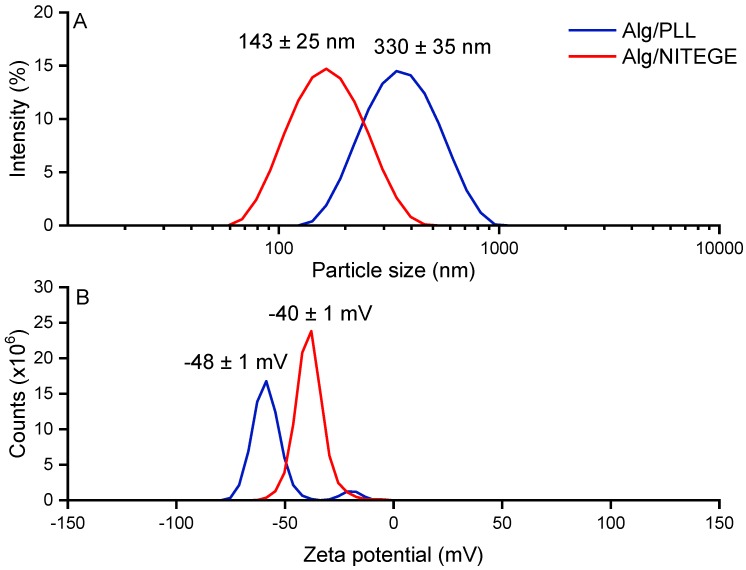
(**A**) Particle size distributions and (**B**) zeta potential curves of alginate/α-poly-L-lysine (Alg/PLL) and alginate/KKKK-GRD-ARGSV↓NITEGE-DRG-KKKK (Alg/NITEGE) nanoparticles in MilliQ, obtained through dynamic light scattering (DLS).

Alg/peptide nanoparticles were obtained via ionotropic gelation, which spontaneously occurs upon combination of aqueous solutions of oppositely charged polyions [26,34]. Solutions of purified sodium alginate and the peptides PLL and KKKK-GRD-ARGSV↓NITEGE-DRG-KKKK (further denoted as NITEGE) were used. Depending on the concentration of the components, we were able to obtain narrowly distributed particles in terms of particle size and zeta potential. The particle formation process was carried out in MilliQ water with 1 mg/mL concentrations of sodium alginate and the peptides each. The corresponding particle size distributions and zeta potential curves are presented in Figure 1. For Alg/PLL, a 4:1 ratio was used, and particles with a size in the range of 330 ± 35 nm and a polydispersity index (PDI) of 0.23 were obtained. The particles had a negative zeta potential of −48 ± 1 mV. However, for the Alg/NITEGE particles, a 1:1 ratio was chosen, leading to an extremely narrow distributed particle size of 143 ± 25 nm and a PDI of 0.15. The zeta potential was found to be −40 ± 1 mV.

Properties like the stability of the nanoparticles or the strength of interaction between the nanoparticles and the surfaces can be estimated from the zeta potential. According to Pujala, sufficient stability of colloidal systems is given at zeta potentials of −30 mV or lower, since coagulation is prevented by the electrostatic repulsion of the particles [35]. Slight changes in the ratio between alginate and the corresponding peptide result in broader distributions of particle sizes and zeta potentials. Since the smaller amount of anionic alginate was added into the formulation of Alg/NITEGE compared to Alg/PLL, the zeta potential of the latter was slightly less negative. However, the difference was lower than expected, probably because of the number of lysine groups, which is reduced by 20% in NITEGE compared to the PLL sequence. Moreover, the presence of anionic amino acids like glutamic acid (E) and aspartic acid (D) further compensates positive charges in the NITEGE peptide.

**Figure 2:**
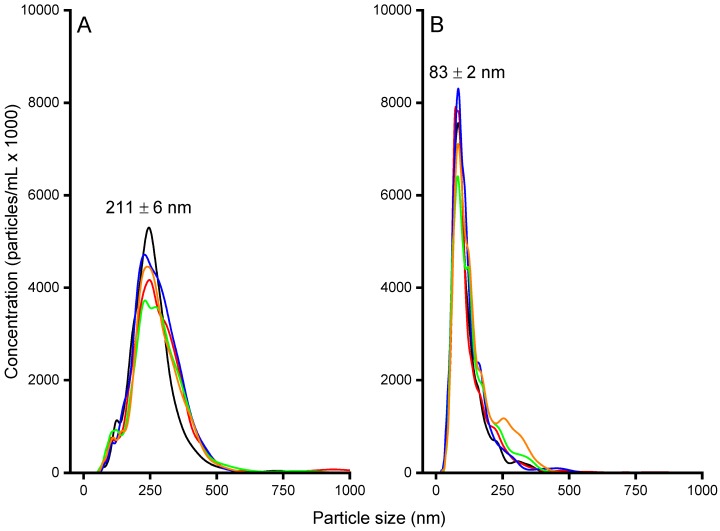
Nanoparticle tracking analysis (NTA) curves for (**A**) Alg/PLL and (**B**) Alg/NITEGE nanoparticles. The particles were prepared according to the standard procedure before the dispersions were diluted by 1:10. Depicted are five different measurements to show the reproducibility.

To investigate particles further, NTA was used, which is a technique of following the Brownian motion of single particles by means of light microscopy. With aid of the Stokes–Einstein relation, the hydrodynamic diameter for each of the tracked particles can be determined. The data obtained for the two Alg/peptide systems are displayed in Figure 2. The NTA measurements resulted in particle sizes of about 211 ± 6 nm for the Alg/PLL system and 83 ± 2 nm for the Alg/NITEGE system, which were somewhat smaller compared to the DLS investigations. This discrepancy can be ascribed to the different evaluation methods of these characterization techniques. Nanoparticle tracking analysis is based on number-weighted size distributions, whereas the primary result of the DLS are intensity-weighted data. Upon conversion of the intensity-weighted size distributions obtained through DLS into number-weighted size distributions, we could elicit hydrodynamic diameters of 208 ± 6 nm for Alg/PLL and 70 ± 15 nm for Alg/NITEGE, respectively. These particle sizes then can be directly correlated to the results obtained from NTA investigations.

### 3.2. Particle Degradation

The degradation of the Alg/peptide nanoparticles in the presence of infection-related proteases is important for the application as a smart drug delivery system. Preliminary investigations with commercially available enzymes were carried out in vitro and monitored via Dynamic Light Scattering. For this purpose, three enzymes were chosen, namely, trypsin, thermolysin, and alginate-lyase. The peptidase trypsin is a mixture of three digestion enzymes cleaving proteins in the gut [36]. It was selected due to its ability to cleave a protein sequence after a lysine residue, as they are present in both the PLL and the NITEGE sequence [37]. Thermolysin is a thermostable enzyme belonging to the family of metallopeptidases. Thermolysin is able to degrade peptide bonds after large and hydrophobic amino acids like leucine, isoleucine, and phenylalanine [38]. Thus, it is not capable to degrade poly-L-lysine, whereas the NITEGE should be hydrolyzed. The effect of the alginate degradation of the particles was investigated through the addition of alginate-lyase. It is an enzyme well known for the cleavage of the glycosidic bond through a β-elimination reaction, generating much smaller molecules, like 4-deoxy-l-erythro-hex-4-enepyranosyluronate [39].

**Figure 3:**
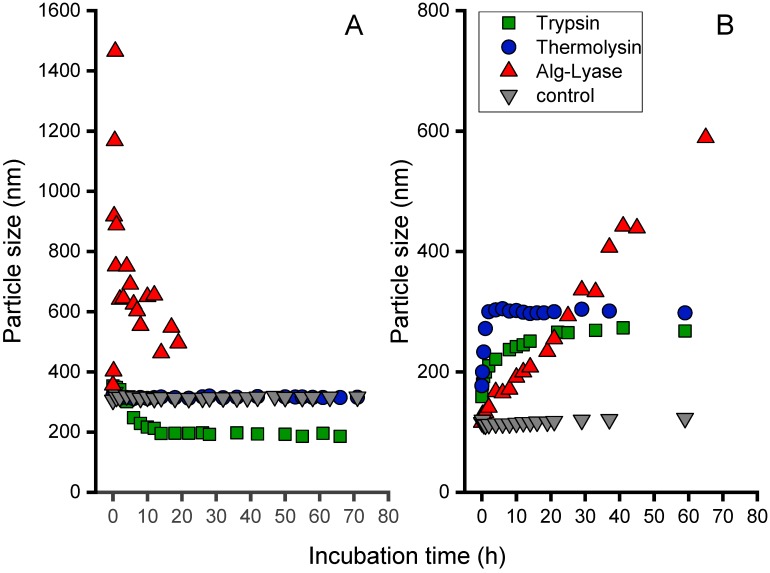
Degradation studies for (**A**) Alg/PLL and (**B**) Alg/NITEGE nanoparticles in MilliQ water (filled triangles) at 37 °C. After formation, the particles were coincubated with enzyme solutions (each 2.5 µg/mL) of thermolysin, trypsin, and alginate-lyase for up to 80 h. The particle size was monitored over the incubation time through DLS.

As shown in Figure 3A, for the Alg/PLL system, no significant change of particle size could be observed in the presence of thermolysin and in the MilliQ water control solution within three days. By contrast, incubation with trypsin led to a decreasing particle size, as expected for a degradation process. The main decrease in particle size took place during the first 20 h. Subsequently, a slower decrease was observed. The particle size was reduced to 50% of their original size. Addition of the lyase, which is capable of cleaving the alginate, induced a particle size increase in the first 4 h and a significant drop afterwards. This behavior is mainly attributed to the aggregation of cleaved alginate residues after the enzymatic reaction. When the particles become too large, a subsequent sedimentation process can be observed, leading to a white precipitate in the cuvette. Thus, the supernatant again exhibits smaller particles to be detected.

The degradation of the Alg/NITEGE system, displayed in Figure 3B, again indicated stable nanoparticles in MilliQ water at 37 °C up to 3 days. Upon addition of trypsin, thermolysin, and alginate-lyase, the particle sizes initially increased for all formulations but to a different extent. The increase in particle size was most prominent for trypsin, as the particles grew to 300% of their original size after 4 h. By contrast, the particle size in the presence of thermolysin and alginate-lyase increased much more slowly, reaching similar size values not before 20 h of incubation time. Unlike trypsin and thermolysin, the addition of alginate-lyase led to a constant rise in particle size with no initial increase in the very first hours. The fact of increasing particle sizes after the addition of enzymes let us surmise that small fragments of the peptides (for trypsin and thermolysin) or alginate (for alginate-lyase) may have been cleaved and reintegrated in the particles. Another explanation is related to particle swelling processes that were reported by McDonald et al. and Thornton et al. as consequences of enzymatic reactions [40,41]. They proposed a particle size increase due to electrostatic repulsion between protonated lysine residues in the peptide sequence and amino groups that form upon cleavage. Nonetheless, a change in particle size upon addition of enzymes indicates that the particles are not stable anymore but will be altered by the proteases in a degradation-like manner. 

Since the Alg/NITEGE nanoparticles were designed to be cleaved by aggrecanase, the influence of this infection-related protease was also investigated. When aggrecanase was added, again, a distinct increase in particle size could be observed, as had been already shown for trypsin and thermolysin, whereas Alg/PLL nanoparticles did not show any change in particle size over 50 h incubation time (data not shown).

### 3.3. Encapsulation of IFN-β

Interferon-β was selected as a model protein. It is an antiviral and antitumoral protein secreted as an immune answer to viral infections like hepatitis or cancer diseases [42]. To determine encapsulation efficiency, a series of known IFN-β concentrations in MilliQ water was prepared and measured by a bioluminescence assay to obtain calibration curve. We encapsulated 5000 U/mL IFN-β in Alg/NITEGE particles through ionic gelation in the presence of the INF-β. The suspension was centrifuged at 16,800 rpm for 15 min. The IFN-β concentration in the supernatant was determined by the bioluminescence assay. The encapsulation efficiency was calculated using the following equation.

$$ee\;\left[\%\right]=\frac{measured\;RLU\;\left(particles+IFN\beta \right)}{measured\;RLU\;\left(free\;IFN\beta \right)}\times 100$$

We could obtain a minimal encapsulation efficiency of about 25 ± 6%. This is significantly lower than the encapsulation efficiency obtained for the growth factor bone morphogenetic protein 2 (BMP-2) in a chitosan/tripolyphosphosphat nanohydrogel (>90%) [30]. The difference can be explained by the complicated interplay of electrostatic and other entropic forces, mediated by van der Waals interactions and hydrogen bonding, which have to be taken into account to understand the binding and release between polymeric carrier and protein [43].

### 3.4. Coating of Titanium Substrates

Titanium and its alloys are among the most popular materials used as orthopedic and dental implants, due to their good mechanical and chemical properties, as well as their biocompatibility [44,45]. Therefore, Ti-6Al-4V alloy plates were used for the coating procedure. The zeta potential of the Alg/peptide nanoparticles was found to be negative (see above). However, the surface charge of titanium has been determined by Kamada et al. to be also negative, with a zeta potential of −25 mV [46]. To facilitate layer-by-layer deposition of the Alg/peptide nanoparticles, adsorption of cationic PEI as a base layer is well established, as it results in a charge reversal of the surface [47]. This base layer was installed by immersion of titanium substrates in an aqueous PEI solution (5% w/w). Onto this base layer, the Alg/peptide suspension was spray-coated. The characterization of the coatings was performed by means of ellipsometry, providing information about the layer thickness, RAIRS, and XPS.

### 3.5. Ellipsometry

The ellipsometric thickness of the PEI-base layer was determined to 3–6 nm with only small deviations. Subsequently, the Alg/peptide dispersions were spray-coated onto the PEI layer for a defined period of time to deposit approximately 20 µL of the nanoparticle dispersion. After a rinsing step, Alg/PLL coatings exhibited film thicknesses in the range of 80 ± 5 nm, while Alg/NITEGE gave lower thicknesses, around 54 ± 6 nm. In contrast to DLS, where hydrodynamic particle sizes were measured, ellipsometry was performed in a dry state. Due to the loss of water, the layer thickness was not expected to be identical to the particle size obtained from DLS measurements. Furthermore, a rearrangement of nanoparticles into homogenous film took place [48]. Aggregation and coalescence of polymer particles from a suspension by evaporation of the solvent is a well-known phenomenon for water-borne paints [49].

Nevertheless, the difference between the hydrodynamic radii for the Alg/peptide nanoparticle could also be found in the layer thicknesses determined by ellipsometry. Additionally, the different zeta potential could have an impact on the layer thicknesses of the Alg/peptide coatings. As mentioned before, the zeta potential is a characteristic feature that describes the stability of the particles, as well as the interaction with the substrate surface. The difference between −48 mV for the Alg/PLL nanoparticles compared to −40 mV for the Alg/NITEGE system is assumed to be caused by the different charge density along the peptide chains. This will also influence the adhesion of the layers on the substrate material. Therefore, Alg/NITEGE coatings, which exhibited a less negative zeta potential, could be partially removed from the substrate surface by rinsing with water.

### 3.6. Characterization of the Coating by Reflection-Absorption Infrared Spectroscopy

We further characterized the layers through RAIRS. In addition to the nanoparticles, both components were coated separately onto titanium substrates and analyzed by RAIRS. In Figure S1 (see Supplementary Material), the relevant wave number range from 2050–900 cm^–1^, which was used to evaluate the deposition of the Alg/peptide particles, is depicted. The PLL spectrum in A showed significant bands, around 1671 cm^–1^ and 1547 cm^–1^, which could be assigned to amide I and amide II bending vibrations [50]. Sodium alginate showed important absorption bands at 1623 cm^–1^ and 1419 cm^–1^, attributed to asymmetric and symmetric stretching vibrations of the carboxylate salt ion, respectively. The band at 1097 cm^–1^ arises from stretching vibrations of the hydroxyl groups present in alginate [51]. In the spectrum of the coated Alg/PLL nanoparticles, all bands aforementioned were detected, suggesting the successful deposition of the nanoparticles on titanium substrates.

Investigations of Alg/NITEGE layers were performed in the same way. The aggrecanase-labile peptide sequence showed distinct bands at 1684 cm^–1^ and 1551 cm^–1^, attributed to the amide I and II bending vibrations, similar to PLL. Further peaks at 1206 cm^–1^ and 1135 cm^–1^ were detected, probably belonging to O-C stretching vibrations from the aspartic acid residues present in the NITEGE sequence. Together with the bands of alginate spectrum, we could again confirm the immobilization of the Alg/NITEGE particles on titanium substrates. 

### 3.7. X-ray Photoelectron Spectroscopy Measurements

**Figure 4:**
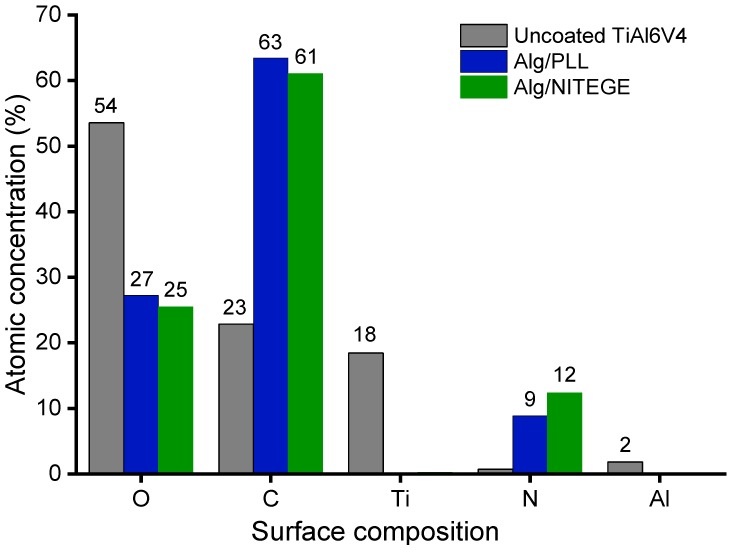
X-ray photoelectron measurement of Alg/peptide coated Ti-6Al-4V substrates and an uncoated Ti-6Al-4V alloy substrate showed the atomic concentration on the surface in percent. Substrates were immersed in polyethyleneimine (PEI) (5% w/w in MilliQ) before Alg/PLL (each 1 mg/mL in MilliQ, volume ratio 4:1), and Alg/NITEGE (each 1 mg/mL, volume ratio 1:1) nanoparticle suspensions were spray-coated onto the surfaces.

The coated substrates were characterized by means of XPS measurements to elucidate the elemental composition of the surface probing the top 10 nm of the film. As can be seen in Figure 4, uncoated titanium samples were compared to the samples coated with Alg/PLL and Alg/NITEGE nanoparticles. For the uncoated surface, high amounts of oxygen (54%) and titanium (18%) could be found, attributed to the passivating titanium oxide layer. The detection of aluminum (2%) was related to the Ti-6Al-4V alloy that was used as substrate material. Additionally, carbon (23%) was detected, which results from common impurities for samples handled in air. By contrast, both Alg/peptide nanoparticle coated samples exhibited no titanium signal, indicating complete coverage of the surfaces with layers > 10 nm. Furthermore, oxygen (26%) was decreased, whilst carbon (62%) and nitrogen (between 9% and 12%) showed a significant increase. 

The atomic concentrations obtained through XPS are in good agreement with the theoretically calculated composition given in Table 1. Thus, the immobilization of Alg/peptide nanoparticles on the titanium surface could be clearly confirmed.

### 3.8. Degradation Behavior (Ellipsometry)

Degradation studies were carried out with substrates, which were coated as described above. In order to evaluate degradation, substrates were incubated at 37 °C in the corresponding enzyme solutions (trypsin, thermolysin, and alginate-lyase with 2.5 µg/mL each) and were investigated by means of ellipsometry with regard to layer thickness in the dry state after defined incubation periods.

**Figure 5:**
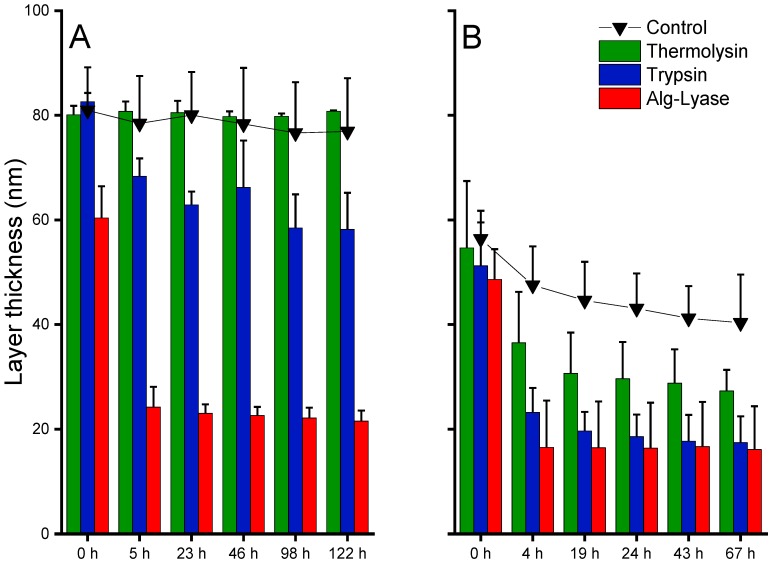
Monitoring ellipsometric layer thicknesses of (**A**) Alg/PLL and (**B**) Alg/NITEGE coatings while coincubated with control, thermolysin, trypsin, and alginate-lyase (each 2.5 µg/mL). *n* = 3.

For the Alg/PLL coatings, as can be seen in Figure 5A, the incubation in MilliQ water as control had no significant effect on the layer thickness of about 80 nm in the respective period. A comparable result was obtained for incubation with thermolysin, indicating stability of the coating against this enzyme. However, a significant decrease in layer thickness was observed when the layer was incubated with trypsin or Alg-lyase. The layers incubated in trypsin solution showed a distinct decrease in layer thickness of 15 nm after the first 5 h, with no further change within the next 122 h. Interestingly, alginate-lyase induced a much stronger degradation in the first 5 h, leading to final thicknesses of about 20 nm.

Similar results could be obtained for the Alg/NITEGE coatings. However, a slightly poorer stability in MilliQ water was observed, as the layer thickness was slowly declining from 60 nm to 40 nm over a period of 67 h. This behavior is probably ascribed to a lower stability of the film, because of the smaller number of lysine groups in the peptide compared to the PLL. Compared to MilliQ conditions, we could observe a more serious degradation of the coatings when incubated in the presence of trypsin and thermolysin. Between these two enzymes, the extent of degradation was more pronounced for trypsin, presumably attributed to the higher number of cleaving sites in the NITEGE peptide. The incubation in alginate-lyase again showed a dramatic decrease after short periods, with no further degradation up to 67 h. Thus, both nanoparticle systems showed sufficient stability in water at 37 °C but got rapidly degraded in the presence of enzymes that can either cleave the peptide crosslinker or the alginate itself. The differences in the layer thickness decrease between thermolysin, trypsin, and alginate-lyase, which could probably be explained by the different cleavage mechanism. 

We suppose the enzymatic reaction leading to the degradation of alginate to be more significant, as can be seen in the decline in layer thickness. Large fragments were degraded in the very first hours and were subsequently removed from the substrates’ surfaces. This process led to the major decrease in layer thickness. After a certain period, the reaction slowed down considerably, because less alginate was present on the substrate.

By contrast, the degradation of the peptide sequences (PLL and NITEGE) seems to play a less prominent part, as the layer thickness only decreased in the range of 15 to 20 nm. While degrading the peptide linker, the alginate remains unaffected and is probably bound to the cationic surface by electrostatic interactions. This process may lead to the masking of the surface by alginate, which cannot be degraded by trypsin or thermolysin. We also observed an influence of the enzyme concentration, where, at a higher enzyme concentration of 50 µg/mL, thickness decrease is more pronounced (see Figure S2, Supplementary Material). Despite all differences, the results clearly indicated that the layers were attached to the titanium substrate and were at least partially degraded by enzymes.

### 3.9. Stability of Deposited IFN-β-Loaded Alg/NITEGE Nanoparticles

**Figure 6:**
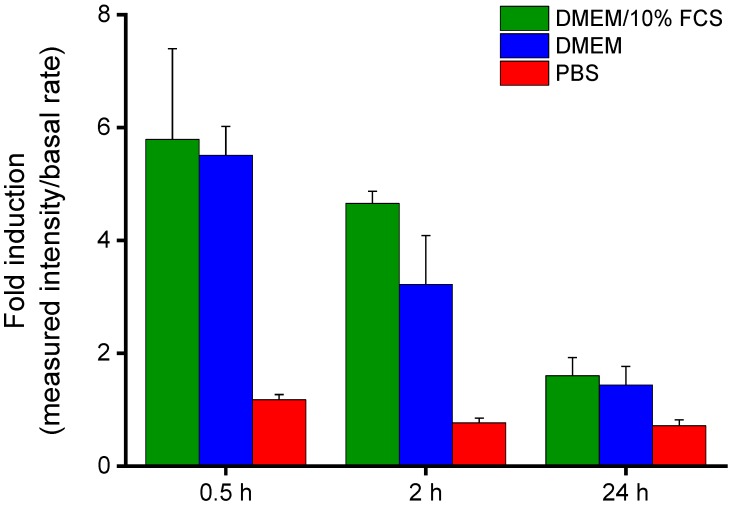
Fold induction values of deposited IFN-β-loaded Alg/NITEGE nanoparticles after coincubation with cell culture medium (DMEM) with and without fetal calf serum (FCS) and phosphate buffered saline (PBS), as reference calculated at three time points.

Aggrecanase-labile IFN-β-loaded Alg/NITEGE nanoparticles (Alg/IFN-β/NITEGE) were deposited onto polished titanium substrates to investigate the release kinetics of the protein. The coated samples were incubated in cell culture media with and without FCS, as well as in PBS. After different times of incubation (30 min, 2 h, and 24 h), the media were removed and the IFN-β concentration was measured using a luciferase assay. Figure 6 shows the fold induction values for each incubation medium after three time points (30 min, 2 h, and 24 h). The fold induction is the bioluminescence intensity measured for the sample divided by the basal intensity that the MX2Luc2 cells exhibit without induction by IFN-β. A fold induction value of 1 indicates that no IFN-β was released. This could be clearly seen for the incubation in PBS, even after 24 h. However, when incubated in a cell culture medium, irrespective of FCS addition, the fold induction values were significantly higher indicating that IFN-β was released. This release might be due to high salt concentrations in DMEM, causing reorganization processes. It is likely that multivalent cations present in DMEM can compete with the NITEGE peptide sequence for the interaction with the alginate. Therefore, an ion exchange would take place that can result in the release of IFN-β. Such a dynamic behavior has been observed for alginate gels by Ionita et al. [52]. Furthermore, a sharp drop could be observed in fold induction between 30 min and 24 h, although there is no media change step. This decrease can be explained by the normal loss of activity of IFN-β when stored in solution. Nevertheless, deposited IFN-β-loaded nanoparticles seemed to be sufficiently stable in PBS, whereas a release in cell culture media must be taken into account.

### 3.10. Cell studies—Cytocompatibility of Nanoparticle Suspensions

**Figure 7:**
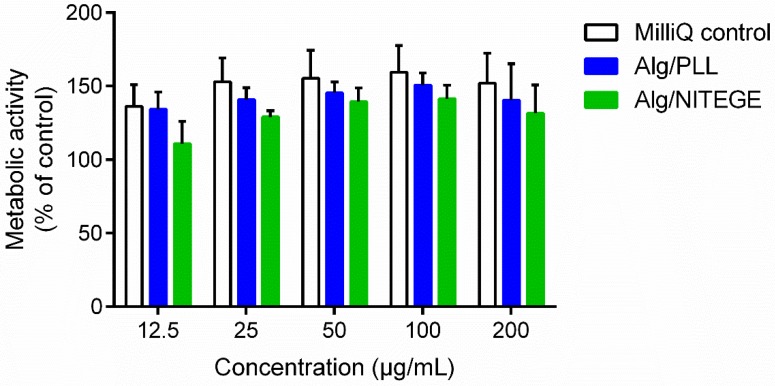
Metabolic activity of human gingival fibroblast (HGFIB) in response to Alg/PLL and Alg/NITEGE nanoparticle suspensions after 24 h. Alg/PLL and Alg/NITEGE nanoparticles and MilliQ water as control were diluted in DMEM with 5% FCS. Untreated cells were set to 100% metabolic activity. Mean and standard deviation of three independent experiments are shown.

The cytocompatibility of the Alg/peptides was studied with HGFIB. Fibroblasts are the most important cell type of soft tissue and are crucial for the wound closure around dental implants [53]. Both a CellTiterBlue assay and a LDH assay were carried out, in order to rule out cytotoxicity of the nanoparticle dispersions. The fluorometric CellTiterBlue assay is based on the ability of living cells to reduce the redox dye resazurin into the fluorescent product resorufin [54]. Human gingival fibroblasts were treated with different concentrations of either the nanoparticle formulations or MilliQ water as control for 24 h. The evaluation of the metabolic activity using CellTiterBlue reagent presented in Figure 7 showed high cell viabilities above 100% independently from the concentration. Thus, there was no negative effect on the metabolic activity of the fibroblasts.

**Figure 8:**
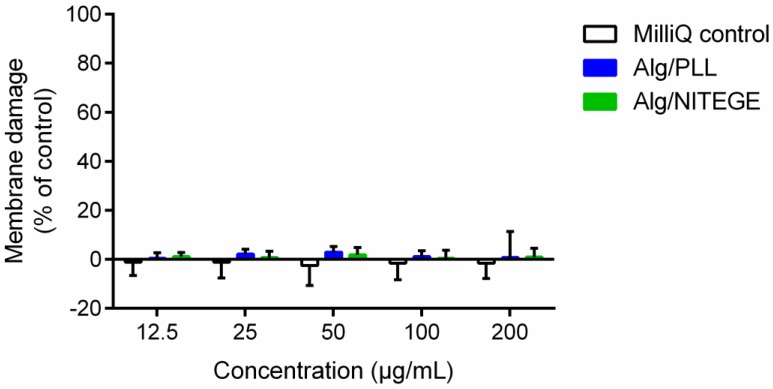
Membrane damage of HGFIB in response to Alg/PLL and Alg/NITEGE nanoparticle suspensions after 24 h. Alg/PLL and Alg/NITEGE nanoparticles and MilliQ water as control were diluted in DMEM with 5% FCS. Untreated cells were set to 0% of membrane damage. Mean and standard deviation of three independent experiments are shown.

Additionally, a colorimetric LDH assay was performed to quantify LDH, which is released in medium by cellular membrane damage, serving as a biomarker for cytotoxicity and cytolysis [54]. Different concentrations of nanoparticle suspensions and MilliQ water as control were tested in combination with human gingival fibroblasts. As shown in Figure 8, no cytotoxic effect was detected over the entire concentration area. Furthermore, in comparison to the MilliQ control, there was only little difference in the percentage of cellular membrane damage for the nanoparticle suspensions. CellTiterBlue as well as LDH assay indicated the nanoparticle suspensions’ excellent cytocompatibility.

### 3.11. Cell Studies—Cell Adhesion/Proliferation on Coated Samples

**Figure 9:**
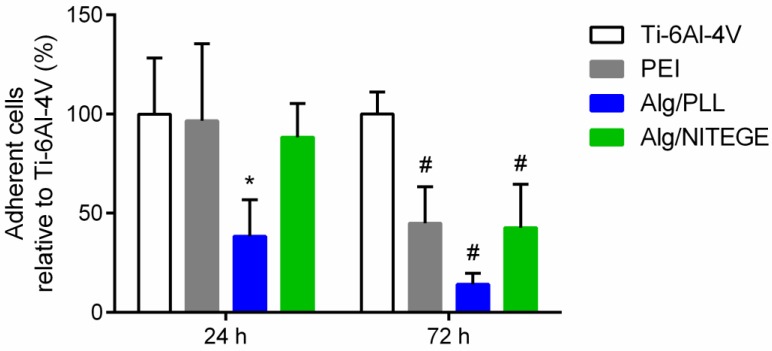
Quantification of adherent HGFIBs on PEI, Alg/PLL and Alg/NITEGE coatings, and uncoated Ti-6Al-4V substrate after 24 and 72 h. The data were related to uncoated Ti-6Al-4V after 24 and 72 h, which were set to 100%. Mean and standard deviation of three independent experiments are shown. The statistical significance was determined using the Kruskal–Wallis test, with *p* = 0.05. * indicates statistical significance (*p* < 0.05) compared to Ti 6Al 4V, 24 h; # indicates statistical significance (*p* < 0.05) compared to Ti-6Al-4V, 72 h.

A modified LDH activity assay was used to investigate the cell adhesion and proliferation of the corresponding coated substrates in comparison to uncoated titanium. In this experiment, the number of cells adherent on the surface was determined by the LDH test after intentional lysis of the cells [33]. In literature, solutions of PEI have been reported to be cytotoxic [47,55]. However, the 3–6 nm thick PEI layer was not expected to have an influence on the cytocompatibility of the coatings, since it was fully covered with the Alg/peptide nanoparticle layer. Both the Alg/peptide coatings and the PEI base layer were examined for cell adhesion. After 24 h incubation, the number of adherent cells was counted, being a measure of the initial cell adhesion. The cell adhesion only on Alg/PLL coating was significantly decreased compared to uncoated Ti-6Al-4V (Figure 9). The cell adhesion on the Alg/NITEGE and PEI coating was similar to the control. After 72 h incubation, the proliferation of the cells on the surfaces could be evaluated. All three coatings exhibited a significant decrease of adherent cells in the region of 15% to 45% compared to the uncoated Ti-6Al-4V. That means the investigated coated surfaces showed cell-repellent properties, because the adhesion and proliferation of the fibroblast cells were reduced. This behavior is well known for pure alginate coatings and is mainly attributed to high hydrophilicity, in addition to the negative charge of the alginate (in Alg/peptide formulations), discouraging protein adsorption and cell attachment [56,57].

**Figure 10:**
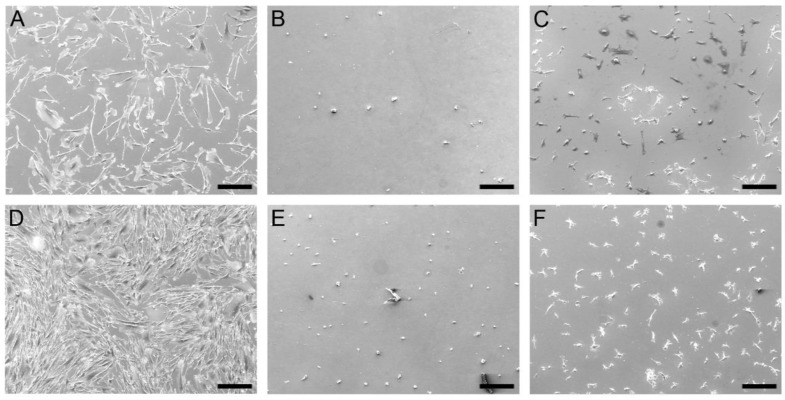
Scanning electron microscopy images of HGFIBs cultured on uncoated Ti-6Al-4V (**A**,**D**), Alg/PLL coated Ti-6Al-4V (**B**,**E**) and Alg/NITEGE coated Ti-6Al-4V (**C**,**F**) after 24 h (**A**–**C**) and 72 h (**D**–**F**). Scale bar = 200 µm.

Scanning electron microscopy images (Figure 10) further confirm these findings. The human gingival fibroblasts showed normal fibroblast morphology and a high proliferation on the uncoated titanium. In contrast, the fibroblast morphology on the Alg/peptide coatings was rounded. On the Alg/PLL coated titanium, only a few rounded cells can be found after 24 and 72 h. The fibroblasts on the Alg/NITEGE coating showed a small spread-out morphology. This rounded morphology indicates a weak surface–cell interaction. The results are in good accordance to the results of the cell adhesion tests (see Figure 9).

In order to overcome the weak adhesion and proliferation of the fibroblasts on the coated surfaces, RGD-modified alginates could be used. The tripeptide RGD, consisting of arginine (R), glycine (G), and aspartic acid (D), is known to promote cell adhesion, since it is a common motive in cells, allowing the binding of surface receptors, like integrins. Preliminary experiments were carried out with RGD-Alginate in combination with the cleavable sequences PLL and NITEGE, which showed a significant improvement of the fibroblast adhesion on the surfaces (Figures S3–S6).

## 4. Conclusions

We have demonstrated that well-defined nanoparticles can be formed via ionotropic gelation of commercially available alginate with cationic peptides. As peptides, poly-l-lysine or an aggrecanase-labile lysine rich sequence (KKKK-GRD-ARGSV↓NITEGE-DRG-KKKK) were used. The nanoparticle dispersions could be used to spray-coat titanium samples, for example, implants, and form homogenous films with less than 80 nm layer thickness. The film formation could be clearly verified by means of ellipsometry, RAIRS, and XPS measurements. Degradation experiments were performed for the dispersed nanoparticles in solution and for deposited layers, showing a sufficient stability in control media and a desired degradation when coincubated with enzymes. Thus, a specific and tunable degradation of the hydrogel network forming the particles in water and the films on the titanium surface was established. The nanoparticle dispersion and films did not show any cytotoxicity towards human gingival fibroblasts. However, the coatings showed weak cell adhesion and almost no cell proliferation. This is a general challenge associated with very hydrophilic alginate hydrogels and could be overcome by binding short RGD peptide sequences to the alginate. Encapsulation of the antiviral agent IFN-β could be realized with moderate efficiency of approximately 25%. The loaded Alg/NITEGE particles also formed thin coatings on titanium. However, in cell culture media, these coatings show limited stability, resulting in a very fast IFN-β-release. A stabilization of the hydrogel network against dynamic cation exchange is necessary.

## Figures and Tables

**Table 1:** Theoretically calculated and by means of X-ray photoelectron spectroscopy (XPS) experimentally determined atomic concentrations of oxygen (O), carbon (C), and nitrogen (N) of the Alg/peptide nanoparticle coatings (each 1 mg/mL in MilliQ, for Alg/PLL volume ratio 4:1 and for Alg/NITEGE volume ratio 1:1). The content of hydrogen was not considered in the calculations. The carbon/nitrogen ratio (C/N ratio) is reported in addition.

	Alg/PLL		Alg/NITEGE	
	Theoretically	Experimentally	Theoretically	Experimentally
Oxygen (O)	33%	27%	28%	25%
Carbon (C)	47%	63%	53%	61%
Nitrogen (N)	5%	9%	12%	12%
C/N-ratio	9.4	7	4.4	5.1

## Supplementary Materials

The following are available online at http://www.mdpi.com/2218-273X/8/4/103/s1, Figure S1: Reflection absorption infrared spectra (RAIRS) of (**A**) titanium coated with PLL, alginate, and the nanoparticle system Alg/PLL and (**B**) titanium coated with NITEGE, alginate, and the nanoparticle system Alg/NITEGE. The relevant wave number range from 2050– 900 cm^–1^ is presented, Figure S2: Monitoring ellipsometric layer thicknesses of (**A**) Alg/PLL and (**B**) Alg/NITEGE↓ARGSV coatings while coincubated with a higher concentration of 50 µg/mL of thermolysin, trypsin, and alginate-lyase. (*N* = 3)., Figure S3: Metabolic activity of HGFIB in response to RGD-Alg/PLL and RGD-Alg/NITEGE nanoparticle suspensions after 24 h. RGD-Alg/PLL and RGD-Alg/NITEGE nanoparticles and MilliQ water as control were diluted in DMEM with 5% FCS. Untreated cells were set to 100% metabolic activity. Mean and standard deviation of three independent experiments are shown, Figure S4: Membrane damage of HGFIB in response to RGD-Alg/PLL and RGD-Alg/NITEGE nanoparticle suspensions after 24 h. RGD-Alg/PLL and RGD-Alg/NITEGE nanoparticles and MilliQ water as control were diluted in DMEM with 5% FCS. Untreated cells were set to 0% of membrane damage. Mean and standard deviation of three independent experiments are shown, Figure S5: Quantification of adherent HGFIBs on RGD-Alg/PLL and RGD-Alg/NITEGE coatings and uncoated Ti-6Al-4V substrate after 24 and 72 h. The data were related to uncoated Ti-6Al-4V after 24 and 72 h, which were set to 100%. Mean and standard deviation of three independent experiments are shown. The statistical significance was determined using the Kruskal–Wallis test, with *p* = 0.05. # indicates statistical significance (*p* < 0.05) compared to Ti-6Al-4V, 72 h, Figure S6: SEM images of HGFIBs cultured on uncoated Ti-6Al-4V (A and D), RGD-Alg/PLL coated Ti-6Al-4V (B and E), and RGD-Alg/NITEGE coated Ti-6Al-4V (C and F) after 24 h (A, B and C) and 72 h (D, E and F). Scale bar = 200 µm.
