# Supplementary material for: Biocompatible Coatings from Smart Biopolymer Nanoparticles for Enzymatically Induced Drug Release
Source: Biomolecules. 2018 Sep 28;8(4):103. doi: 10.3390/biom8040103 (PMC6315368; doi:10.3390/biom8040103)
Supplement: Supplementary file 1 [file biomolecules-08-00103-s001.pdf]

Supplementary Material

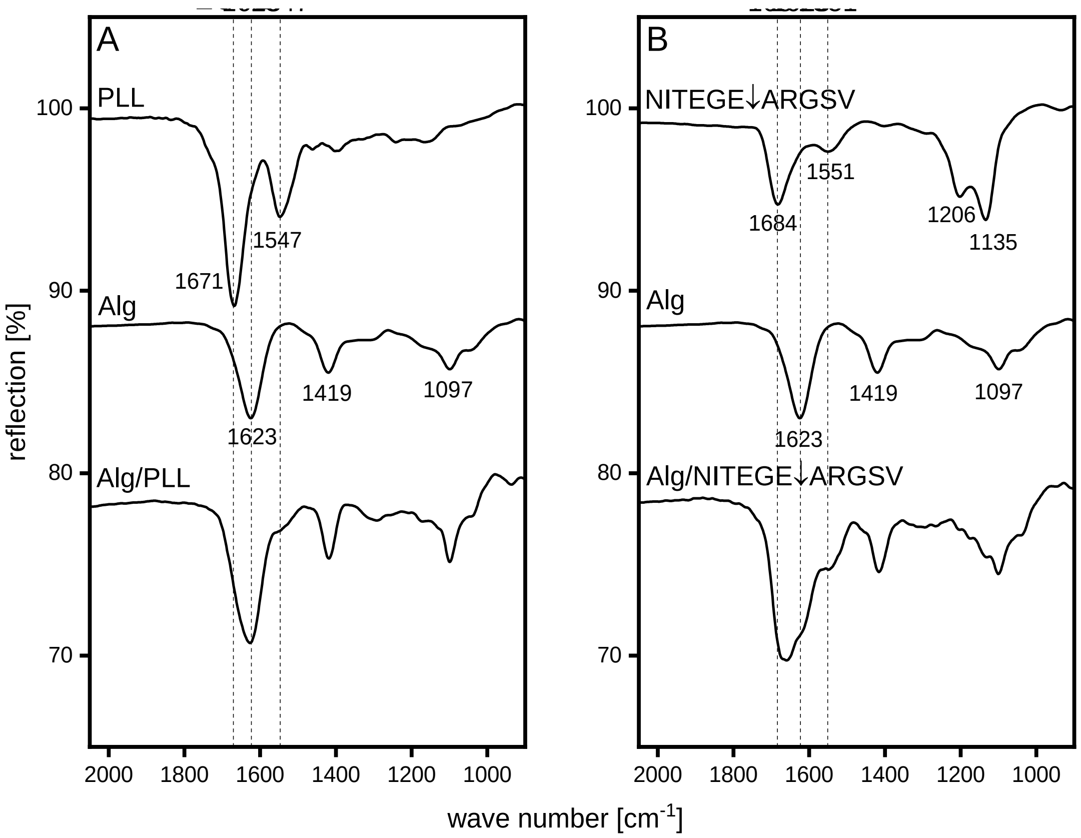

Figure S1: Reflection absorption infrared spectra (RAIRS) of A) titanium coated with PLL, alginate and the nanoparticle system Alg/PLL and B) titanium coated with NITEGE, alginate and the nanoparticle system Alg/NITEGE. The relevant wave number range from 2050 - 900 cm-1 is presented.

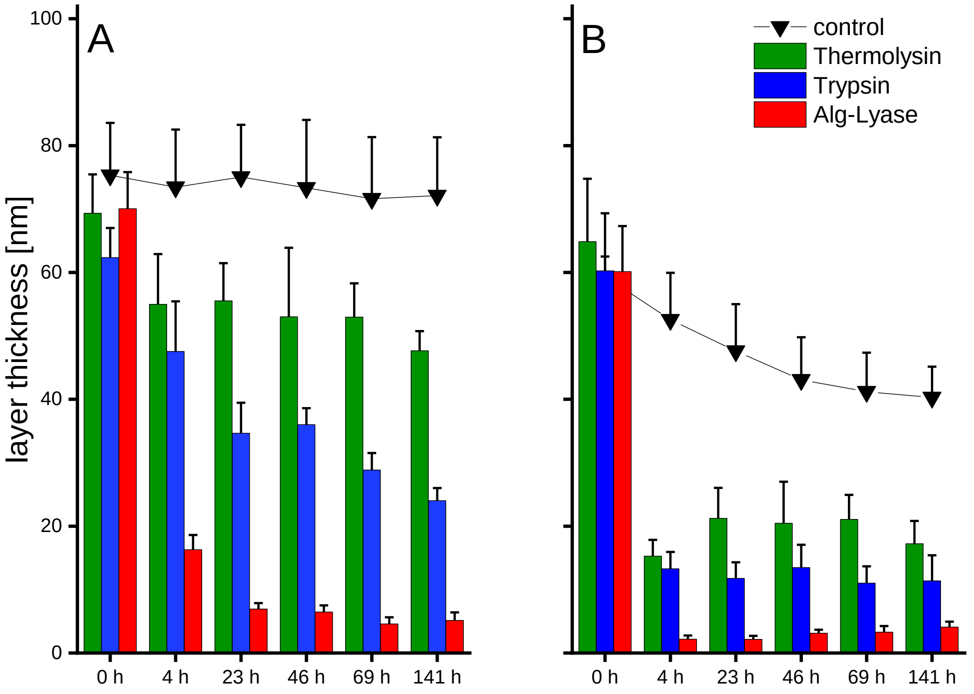

Figure S2: Monitoring ellipsometric layer thicknesses of A) Alg/PLL and B) Alg/NITEGE↓ARGSV coatings while coincubated with a higher concentration of 50 µg/mL of thermolysin, trypsin and alginate-lyase. (*n* = 3).

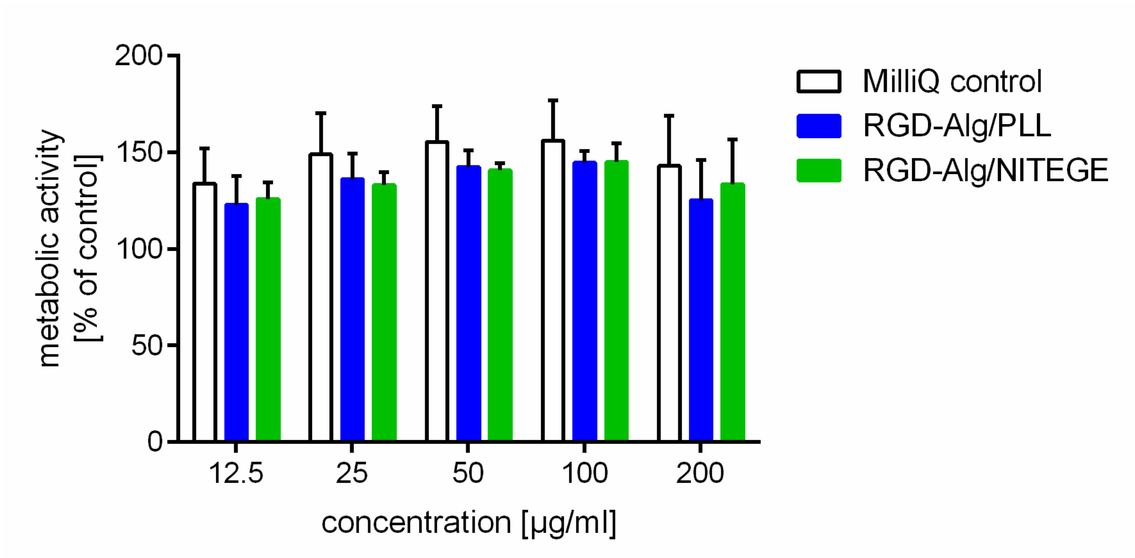

Figure S3: Metabolic activity of HGFIB in response to RGD-Alg/PLL and RGD-Alg/NITEGE nanoparticle suspensions after 24 hours. RGD-Alg/PLL and RGD-Alg/NITEGE nanoparticles and MilliQ water as control were diluted in DMEM with 5% FCS. Untreated cells were set to 100% metabolic activity. Mean and standard deviation of three independent experiments are shown.

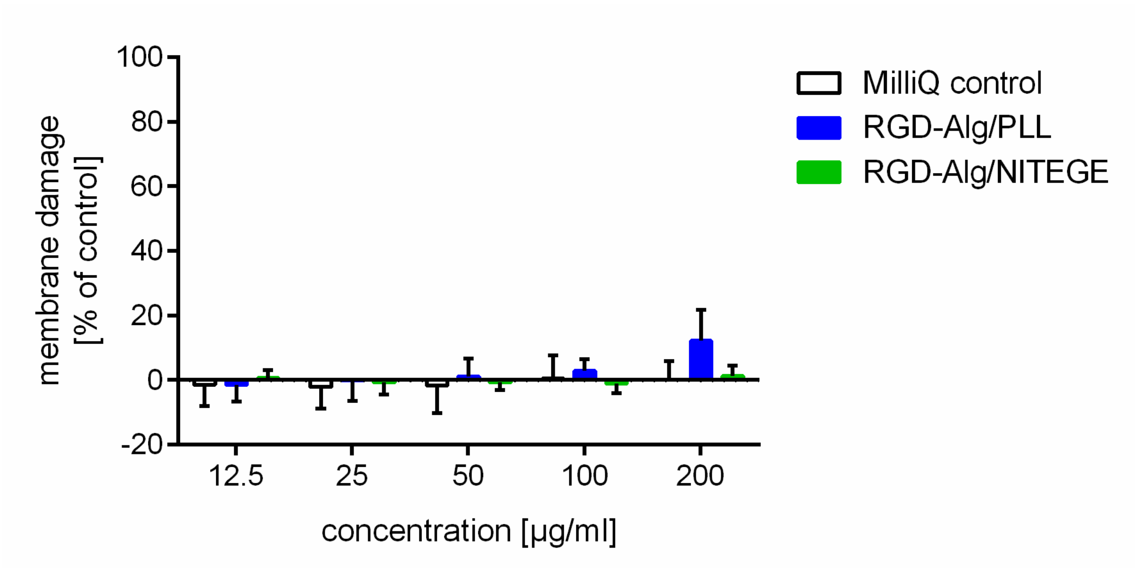

Figure S4: Membrane damage of HGFIB in response to RGD-Alg/PLL and RGD-Alg/NITEGE nanoparticle suspensions after 24 hours. RGD-Alg/PLL and RGD-Alg/NITEGE nanoparticles and MilliQ water as control were diluted in DMEM with 5% FCS. Untreated cells were set to 0% of membrane damage. Mean and standard deviation of three independent experiments are shown.

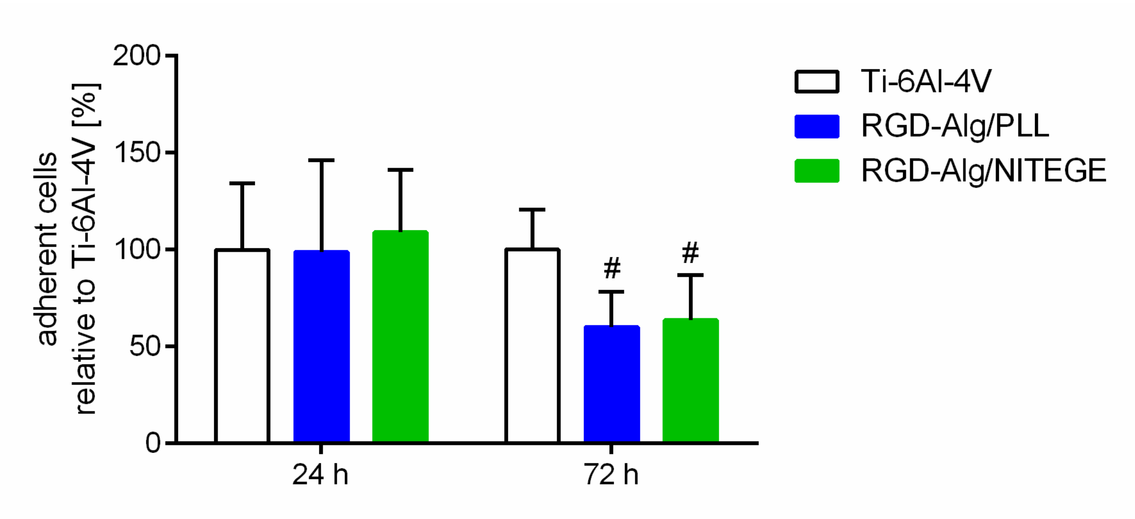

Figure S5: Quantification of adherent HGFIBs on RGD-Alg/PLL and RGD-Alg/NITEGE coatings and uncoated Ti-6Al-4V substrate after 24 and 72 hours. The data were related to uncoated Ti-6Al-4V after 24 and 72 hours which were set to 100%. Mean and standard deviation of three independent experiments are shown. The statistical significance was determined using the Kruskal-Wallis test with $p = 0.05$. # indicates statistical significance ($p < 0.05$) compared to Ti-6Al-4V, 72 hours.

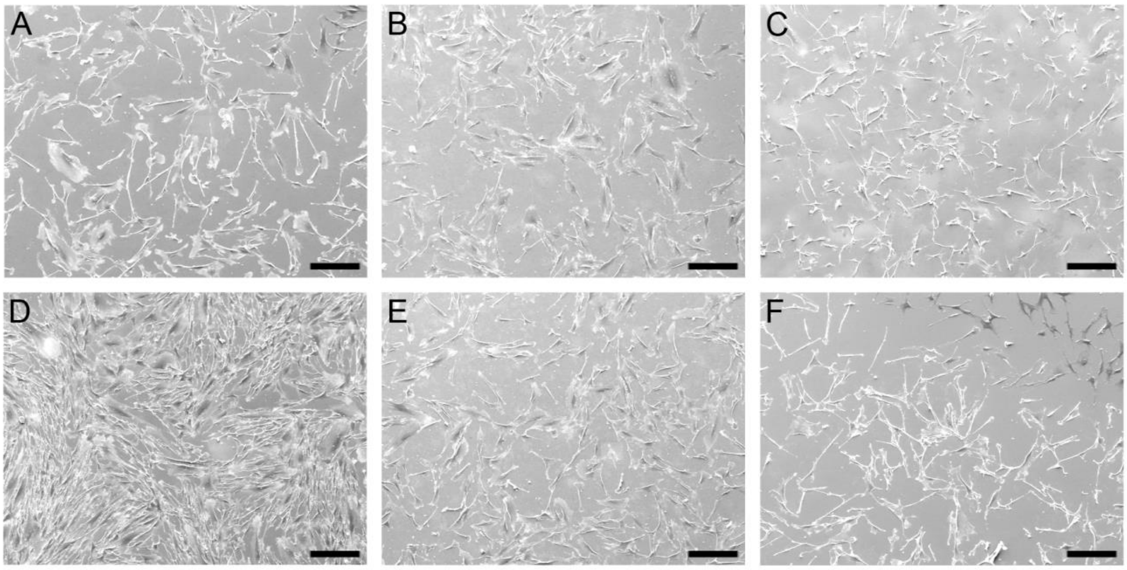

Figure S6: SEM images of HGFIBs cultured on uncoated Ti-6Al-4V (A and D), RGD-Alg/PLL coated Ti-6Al-4V (B and E) and RGD-Alg/NITEGE coated Ti-6Al-4V (C and F) after 24 hours (A, B and C) and 72 hours (D, E and F). Scale bar = 200 μm.
